# College and Hospital Notes

**Published:** 1905-01

**Authors:** 


### COLLEGE AND HOSPITAL NOTES.

The Michigan College of Medicine and Surgery is erecting a new building at the southwest corner of Second and Porter streets, Detroit, the corner stone of which was laid December 13, 1904. The Journal acknowledges the receipt of an invitation to attend the ceremonies.

The Lincoln Hospital, East 141st street and Southern Boulevard, New York, through its managers, announces that Dr. Louis Faugeres Bishop will give a series of clinical lectures on general medical diagnosis in the wards of the hospital on Wednesday afternoons, commencing December 14, 1904, at 3 o'clock. The course will be free to the medical profession.

